# CT-based radiomics with various classifiers for histological differentiation of parotid gland tumors

**DOI:** 10.3389/fonc.2023.1118351

**Published:** 2023-03-10

**Authors:** Yang Lu, Haifeng Liu, Qi Liu, Siqi Wang, Zuhui Zhu, Jianguo Qiu, Wei Xing

**Affiliations:** Radiology, Third Affiliated Hospital of Soochow University, Changzhou, Jiangsu, China

**Keywords:** radiomics, computed tomography, tumor, differentiation, parotid gland (PG)

## Abstract

**Objective:**

This study assessed whether radiomics features could stratify parotid gland tumours accurately based on only noncontrast CT images and validated the best classifier of different radiomics models.

**Methods:**

In this single-centre study, we retrospectively recruited 249 patients with a diagnosis of pleomorphic adenoma (PA), Warthin tumour (WT), basal cell adenoma (BCA) or malignant parotid gland tumours (MPGTs) from June 2020 to August 2022. Each patient was randomly classified into training and testing cohorts at a ratio of 7:3, and then, pairwise comparisons in different parotid tumour groups were performed. CT images were transferred to 3D-Slicer software and the region of interest was manually drawn for feature extraction. Feature selection methods were performed using the intraclass correlation coefficient, *t* test and least absolute shrinkage and selection operator. Five common classifiers, namely, random forest (RF), support vector machine (SVM), logistic regression (LR), K-nearest neighbours (KNN) and general Bayesian network (Gnb), were selected to build different radiomics models. The receiver operating characteristic curve, area under the curve (AUC), accuracy, sensitivity, specificity and F-1 score were used to assess the prediction performances of these models. The calibration of the model was calculated by the Hosmer–Lemeshow test. DeLong’s test was utilized for comparing the AUCs.

**Results:**

The radiomics model based on the RF, SVM, Gnb, LR, LR and RF classifiers obtained the highest AUC in differentiating PA from MPGTs, WT from MPGTs, BCA from MPGTs, PA from WT, PA from BCA, and WT from BCA, respectively. Accordingly, the AUC and the accuracy of the model for each classifier were 0.834 and 0.71, 0.893 and 0.79, 0.844 and 0.79, 0.902 and 0.88, 0.602 and 0.68, and 0.861 and 0.94, respectively.

**Conclusion:**

Our study demonstrated that noncontrast CT-based radiomics could stratify refined pathological types of parotid tumours well but could not sufficiently differentiate PA from BCA. Different classifiers had the best diagnostic performance for different parotid tumours. Our study findings add to the current knowledge on the differential diagnosis of parotid tumours.

## Introduction

Parotid gland tumours are the main tumours of the salivary glands, and more than 80% are benign. However, an early accurate diagnosis is still needed to define the proper surgical treatment (1). For patients with malignant parotid gland tumours (MPGTs), total parotidectomy is necessary, and postoperative chemoradiation is considered if patients have high-risk factors (2). Among benign parotid gland tumours (BPGTs), the major types are Warthin tumour (WT), pleomorphic adenoma (PA) and basal cell adenoma (BCA), and the operation types are also different. Due to its higher malignancy and recurrence rates, PA is treated by partial parotidectomy (3), while WA and BCA are treated only by local surgical excision of the tumour or by conservative treatment, given that malignant transformation is rare (4).

Thus, a simple and effective diagnostic method is crucial and necessary for the differential diagnosis of parotid tumours before surgical treatment. Routine fine needle aspiration is largely dependent on the experience of the clinical operators, as the diagnostic accuracy is sometimes poor due to insufficient or nonrepresentative aspiration (5). In addition, the conventional radiological features of different parotid tumour types may considerably overlap (6). Some studies have reported that changes in parotid tumour margins may not indicate malignancy, and heterogeneously enhanced features cannot be used to distinguish benign from malignant parotid tumours (7, 8). Some BPGTs resemble MPGTs with a heterogeneous appearance due to the existence of the area of cystoid variation and necrosis (9). All of these results present significant diagnostic challenges in the preoperative diagnosis of parotid gland tumours.

Radiomics is a fast-growing research field that is widely used in tumour imaging. The radiomics approach can automatically extract comprehensive data present in imaging modalities and uncover much more quantitative tumour information than our eyes can detect. In recent years, multiple studies have reported that radiomics may be applied to parotid gland tumours with promising preoperative diagnostic results (10). Li et al. confirmed that radiomics analysis of ultrasound images may help improve the discrimination of BPGTs from MPGTs (11). Zheng et al. developed a computed tomography (CT)-based radiomics nomogram to distinguish benign lymphoepithelial lesions from mucosa-associated lymphoid tissue lymphoma, which has promising predictive efficacy (12). In addition, the magnetic resonance (MR) radiomics model has yielded excellent diagnostic performance in differentiating BPGTs from MPGTs and PA from WT (13–18).

Many studies have explored radiomics for the differential diagnosis of parotid tumours based on multiphasic CT or multisequence MR radiomics features; however, it is still necessary to further explore the diagnostic performance of radiomics models based on noncontrast CT. Contrast-enhanced CT or MR studies have superior diagnostic results. However, they often have downsides, and MR may require long acquisition times and have absolute and relative MR contraindications. Contrast-enhanced CT may often burden the patient with more radiation exposure and have contrast agent contraindications. These factors could make noncontrast CT-based radiomics an attractive choice, at least in selected patients. Another potential advantage of CT-based radiomics is the possibility of detecting and characterizing incidental parotid masses in patients undergoing CT for other unrelated reasons. Furthermore, previous CT radiomics studies focused on distinguishing benign from malignant parotid tumours, but there is little research addressing the possibility of distinguishing among the detailed pathological types of parotid tumour. Typically, only a single machine learning classifier was used in previous research, and different classifiers may lead to different diagnostic performances. Hence, it would be beneficial to evaluate whether noncontrast CT-based radiomics can perform well in stratifying different pathological types of parotid tumours and whether there are differences in the diagnostic value of various machine learning classifiers in the diagnosis of parotid gland tumours. This may help distinguish different parotid tumours accurately and conveniently and guide the selection of the best model for future multicentre research of large datasets.

The purpose of this study was to construct different radiomics models based on noncontrast CT images with five mainstream classifiers to compare the predictive ability of various radiomics models for different parotid tumours, such as MPGTs, PA, WT and BCA, and to determine the classifier with the best diagnostic performance for each parotid tumour.

## Materials and methods

### Patients

**Figure 1:**
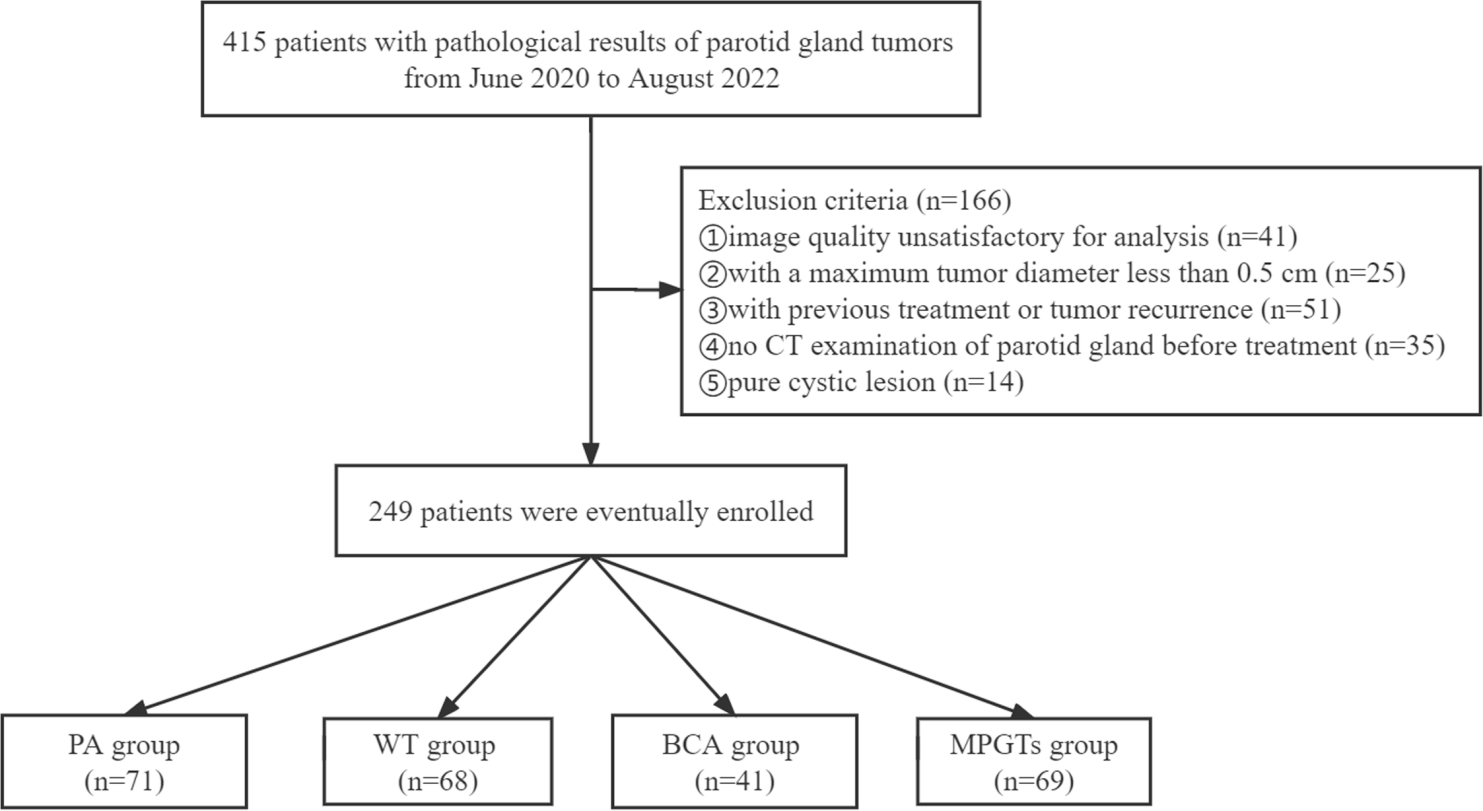
Flowchart for selecting the study population. PA, pleomorphic adenoma; WT, Warthin tumour; BCA, basal cell adenoma; MPGTs, malignant parotid gland tumours.

In this single-centre retrospective study, a total of 415 patients with definite pathological results indicating a parotid gland tumour in the Third Affiliated Hospital of Soochow University were registered from June 2020 to August 2022. The exclusion criteria were as follows (1): parotid tumour recurrence or previous treatment (n=51) (2); no CT examination of the parotid gland before treatment (n=35) (3); maximum tumour diameter less than 0.5 cm (n=25) (4); unsatisfactory image quality due to the existence of metallic or beam hardening artefacts (n=41); or (5) simple cystic lesions (n=14). Thus, a total of 249 patients were included in our study. The baseline clinical characteristics were collected by retrieving the patients’ hospital records. CT was performed with four CT scanners: a double source scanner (SOMATOM Definition Flash, Siemens Healthcare, Forchheim, Germany), a 64-slice CT scanner (Discovery 750 HD, GE Healthcare, Milwaukee, Wisconsin), a 320-slice CT scanner (Aquilion ONE, Toshiba Medical Systems, Otawara, Japan), and a 256-slice CT scanner (Brilliance iCT; Philips Healthcare, Cleveland, OH, USA). According to the pathological results of their parotid gland tumours, the patients were divided into the MPGT, PA, WT and BCA groups. The flowchart for selecting the study population is shown in **Figure 1**. Our study was approved by the ethics committee of the Third Affiliated Hospital of Soochow University, Jiangsu, China, and exempted from informed consent requirements due to the retrospective nature of the study.

### CT image acquisition

Each patient underwent noncontrast imaging with a multislice spiral CT scanner. The CT scanners and parameters were as follows (1): Discovery 750 HD: 120 kV tube voltage; smart mA (100-450 mAs) tube current, section thickness, 2.5 mm; section interval, 2.5 mm; gantry rotation time, 0.6 seconds; detector collimation, 64 mm × 0.625 mm; matrix512×512 (2); SOMATOM Definition Flash: 120 kV tube voltage; tube current with dose modulation (Care Dose 4D), section thickness, 3 mm; section interval, 3 mm; gantry rotation time, 0.5 seconds; detector collimation, 128 mm × 0.6 mm; matrix512×512 (3); Aquilion ONE: 120 kV tube voltage; 250 mAs tube current, section thickness, 3 mm; section interval, 3 mm; gantry rotation time, 0.35 seconds; detector collimation, 320 mm × 0.5 mm; matrix512×512 (4); Brilliance iCT: 120 kV tube voltage; 250 mAs tube current, section thickness, 3 mm; section interval, 3 mm; gantry rotation time, 0.27 seconds; detector collimation, 256 mm × 0.625 mm; matrix512×512. All scans were performed from 1 cm below the aortic arch to the top of the head.

### ROI segmentation

All noncontrast CT images were stored in the Digital Imaging and Communications in Medicine format and imported to 3D-Slicer software for manual segmentation of the regions of interest (ROIs) by two radiologists who were blinded to the pathological results. Contours were drawn slice-by-slice within the borders of the tumours on axial CT images, excluding adjacent bone and vessels. The intraclass correlation coefficients (ICCs) were used to evaluate the stability and agreement of the features, and an ICC greater than 0.75 indicated good agreement.

### Imaging feature extraction

Image preprocessing and feature extraction were performed using the open-source package PyRadiomics 3.0 in python software (version 3.7.6; http://www.radiomics.io/pyradiomics.html). To eliminate the potential impact of the different CT devices on the extracted features, a voxel spacing of 1 × 1 × 1 mm³ was performed to resample the images, and a fixed bin width of 25 was used to normalize image intensity (19). Then, 1323 features were retrieved from each VOI as follows: (a) shape-based features; (b) first-order statistics features; (c) grey-level co-occurrence matrix-based features (GLCM); (d) grey-level run-length matrix-based features (GLRLM); (e) grey-level size zone matrix (GLSZM); (f) neighbouring grey tone difference matrix (NGTDM); (g) grey-level dependence matrix (GLDM) and (h) transform-filtered features (including square, square root, logarithm, exponential, gradient, Laplacian of Gaussian [LOG], wavelet). Finally, z score normalization was also performed for all features to reduce the influence of different dimensions among features (20).

### Feature selection

In this study, patients were divided into four different groups (PA, WT, BCA and MPGT) according to pathological type. In addition, each patient was randomly assigned to the training or test cohort at a ratio of 7:3, and then, pairwise comparisons were performed between different groups after analysis was performed according to the following pipeline. Three steps were performed for feature selection. First, the features with ICCs >0.75 were selected due to their stability. Second, to select features that differed significantly between groups, the *t test* was performed. Finally, a least absolute shrinkage and selection operator (LASSO) regression model with 10-fold cross-validation was performed to select features with nonzero coefficients.

### Statistical analysis

**Figure 2:**
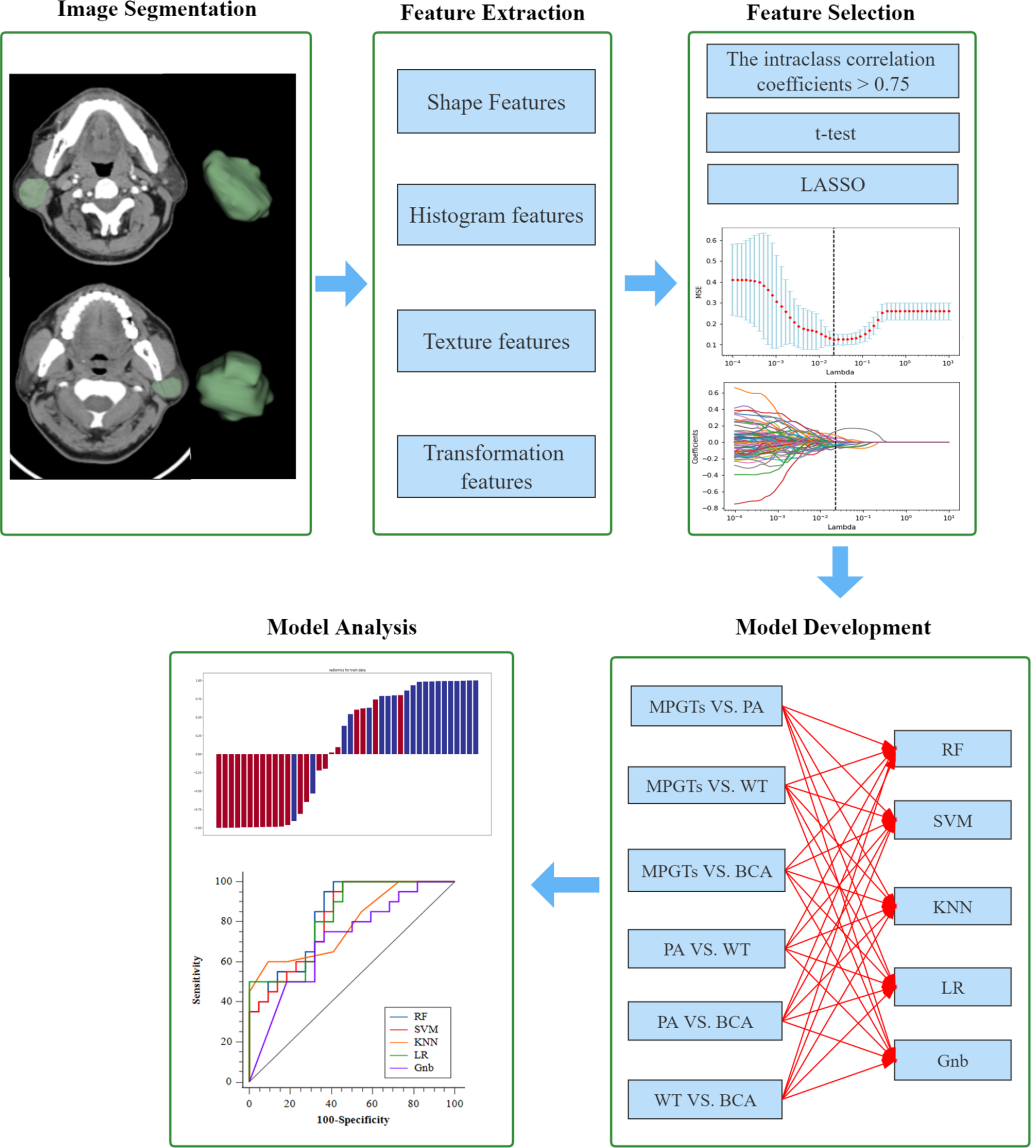
Workflow of the radiomics analysis. LASSO, least absolute shrinkage and selection operator; MPGTs, malignant parotid gland tumors; PA, pleomorphic adenoma; WT, warthin tumor; BCA, basal cell adenoma; SVM, support vector machine; RF, random forest; KNN, k-Nearest Neighbor; LR, logistic regression; Gnb, GaussianNB.

The final selected features were utilized for modelling with five mainstream classifiers, including logistic regression (LR), K-nearest neighbours (KNN), support vector machine (SVM), random forest (RF) and GaussianNB (Gnb). The diagnostic performance of each model for the differential diagnosis of parotid gland tumours (PA and MT, PA and WT, PA and BCA, WT and MT, WT and BCA, and BCA and MT) was quantitatively evaluated by means of the area under the curve (AUC) of the receiver operating characteristic (ROC), accuracy, sensitivity, specificity and F-1 score. The calibration of the radiomics model was calculated by the Hosmer–Lemeshow test. DeLong’s test was utilized for comparisons of AUCs. A *p* value < 0.05 indicates a significant difference. The distributions of radiomics scores for each validation cohort patient in the different models are presented as a waterfall plot. All the above processes were implemented in Python (version 3.7.6), except DeLong’s test, which was implemented with MedCalc19.8 software (MedCalc, Ostend, Belgium). A flow diagram describing the radiomics analysis process is shown in **Figure 2**.

## Results

### Study cohort

Among the 249 patients included in this study, 154 (61.85%) were men, and 95 (38.15%) were women. The average age of the patients was 52.72 ± 15.22 years. Among the 180 BPGT cases, the most common subtype was PA (71, 39.44%), followed by WT (68, 37.78%) and BCA (41, 22.78%). The other 69 lesions were MPGTs. The numbers of cases evaluated with the Discovery 750 HD, SOMATOM Definition Flash, Aquilion ONE and Brilliance iCT scanners were 61, 75, 71 and 42, respectively.

### MPGTs *vs.* PA

In the comparisons of MPGTs and PAs, a total of 503 radiomics features were selected after being screened by the ICC and *t test*. Then, 16 features were finally selected by LASSO for building the radiomics models, and the best tuned regularization parameter lambda was 0.0569. There were 1 first-order statistics feature, 1 GLCM feature, 1 gradient feature and 13 wavelet features among the final selected features.

**Figure 3:**
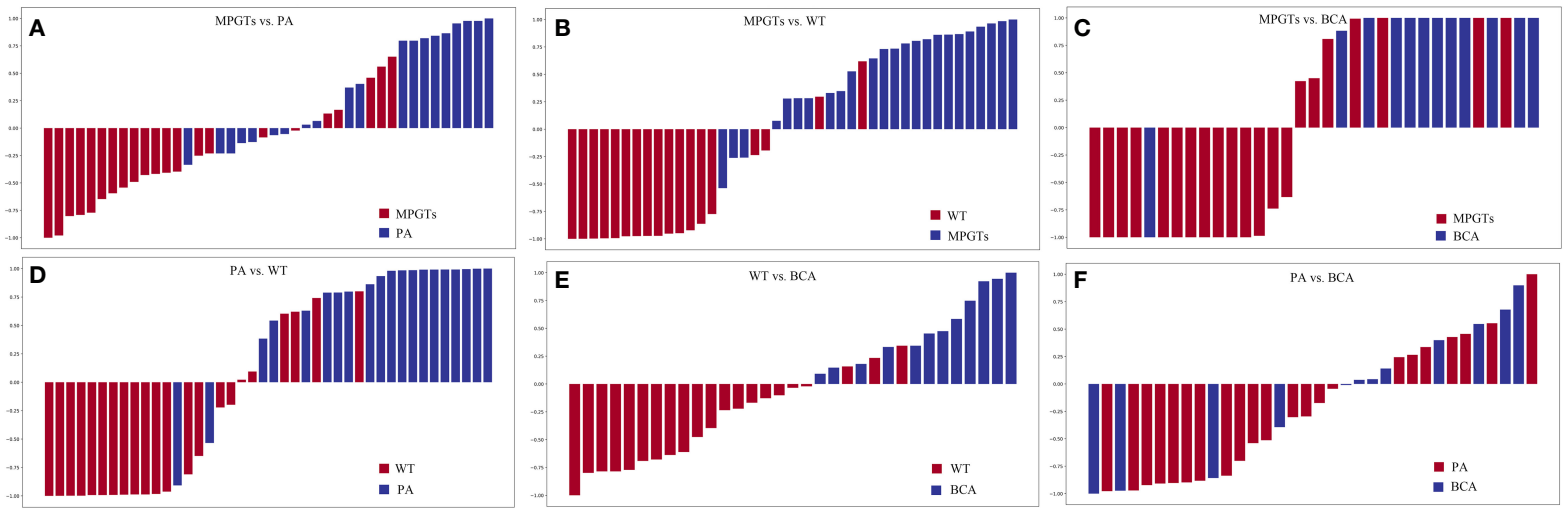
Waterfall plots for distribution of scores based on different radiomics models for each patient in the validation cohort. **(A)** MPGTs vs. PA; **(B)** MPGTs vs. WT; **(C)** MPGTs vs. BCA; **(D)** PA vs. WT; **(E)** WT vs. BCA; **(F)** PA vs. BCA. MPGTs, malignant parotid gland tumors; PA, pleomorphic adenoma; WT, warthin tumor; BCA, basal cell adenoma.

**Figure 4:**
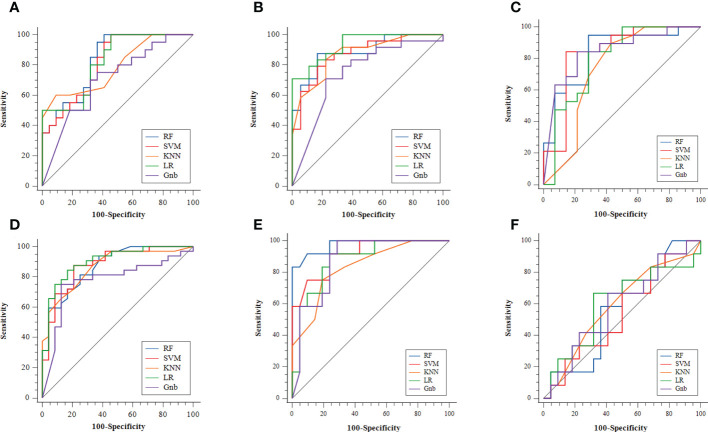
The ROC curves of the different radiomics models: **(A)** MPGTs *vs.* PA; **(B)** MPGTs *vs.* WT; **(C)** MPGTs *vs.* BCA; **(D)** PA *vs.* WT; **(E)** WT *vs.* BCA; **(F)** PA *vs.* BCA. MPGTs, malignant parotid gland tumors; PA, pleomorphic adenoma; WT, warthin tumor; BCA, basal cell adenoma; SVM, support vector machine; RF, random forest; KNN, k-Nearest Neighbor; LR, logistic regression; Gnb, GaussianNB.

The radiomics model of the RF classifier obtained the best diagnostic performance in differentiating PA from MPGTs compared with the other four classifiers. The AUC and accuracy were 0.834 and 0.71, with sensitivity, specificity and F-1 scores of 0.87, 0.62 and 0.82, respectively. The *p* value of the RF model in the Hosmer–Lemeshow test was 0.139 (>0.05), so the calibration of the RF model was reliable. Analysis by Delong’s test showed that the AUC of the RF model was the highest but was significantly higher than that of the Gnb model only (*p*=0.021), with no significant differences compared to those of the other three models (*p>*0.05). The waterfall plot of the RF model in differentiating PA from MPGTs in the validation cohort is presented in **Figure 3A**. The ROC curve is shown in **Figure 4A**.

### MPGTs *vs.* WT

In the differentiation between MPGTs and WTs, a total of 456 radiomics features were selected according to the ICC and *t test*. Then, 14 features were finally selected by LASSO for building the radiomics models, and the best tuned regularization parameter lambda was 0.0281. There were 1 shape-based feature, 1 exponential feature, 1 logarithm feature and 11 wavelet features among the final selected features.

The radiomics model of the SVM classifier had the best diagnostic performance in differentiating WT from MPGTs compared with the other four classifiers. The AUC and accuracy were 0.893 and 0.79, with sensitivity, specificity and F-1 values of 0.79, 0.78 and 0.84, respectively. The *p* value of the RF model in the Hosmer–Lemeshow test was 0.911 (>0.05), so the calibration of the SVM model was reliable. Analysis by Delong’s test showed that the AUC of the SVM model was significantly better than that of the LR model (*p*=0.022) or Gnb model (*p*=0.010), but there was no significant difference compared to the AUCs of the RF and KNN models (*p>*0.05). The waterfall plot of the SVM model in differentiating WT from MPGTs in the validation cohort is presented in **Figure 3B**. The ROC curve is shown in **Figure 4B**.

### MPGTs *vs.* BCA

In the differential diagnosis between MPGTs and BCAs, a total of 503 radiomics features were selected after being screened by the ICC and *t test*. Then, 16 features were finally selected by LASSO for building the radiomics models, and the best tuned regularization parameter lambda was 0.036. There were 1 shape-based feature, 1 GLCM feature, 1 GLRLM feature, 2 exponential features and 11 wavelet features among the final selected features.

The radiomics model of the Gnb classifier obtained the best diagnostic performance in differentiating BCA from MPGTs compared with the other four classifiers. The AUC and accuracy were 0.844 and 0.79, with sensitivity, specificity and F-1 values of 0.84, 0.79 and 0.84, respectively. The *p* value of the Gnb model in the Hosmer–Lemeshow test was 0.908 (>0.05), so the calibration of the Gnb model was reliable. Analysis by DeLong’s test showed that the Gnb model achieved the highest AUC, but there were no significant differences between the AUC of the Gnb model and those of the other four models (*p>*0.05). The waterfall plot of the Gnb model in differentiating BCA from MPGTs in the validation cohort is presented in **Figure 3C**. The ROC curve is shown in **Figure 4C**.

### PA *vs.* WT

In the comparisons of PAs and WTs, a total of 336 radiomics features were selected after being screened by the ICC and *t test*. Then, 18 features were finally selected by LASSO for building the radiomics models, and the best tuned regularization parameter lambda was 0.022. There were 2 shape-based features, 1 first-order statistics feature, 1 GLCM feature, 1 gradient feature, 3 logarithm features, 2 square root features and 8 wavelet features among the final selected features.

Compared with the other four classifiers, the radiomics model of the LR classifier obtained the best diagnostic performance in differentiating PA from WT. The AUC and accuracy were 0.902 and 0.88, with sensitivity, specificity and F-1 values of 0.84, 0.83 and 0.86, respectively. The *p* value of the LR model in the Hosmer–Lemeshow test was 0.243 (>0.05), so the calibration of the LR model was reliable. Analysis by Delong’s test showed that the LR model achieved the highest AUC but that the AUC was significantly higher than that of the Gnb model only (*p*=0.019), with no significant differences compared to those of the other models (*p>*0.05). The waterfall plot of the LR model in differentiating PA from WT in the validation cohort is presented in **Figure 3D**. The ROC curve is shown in **Figure 4D**.

### WT *vs.* BCA

In the differential diagnosis between WTs and BCAs, a total of 193 radiomics features were selected after being screened by the ICC and *t test*. Then, 15 features were finally selected by LASSO for building the radiomics models, and the best tuned regularization parameter lambda was 0.028. There were 1 shape-based feature, 2 first-order statistics features, 1 gradient feature, 1 logarithmic feature, 1 square root feature and 9 wavelet features among the final selected features.

The radiomics model of the RF classifier obtained the best diagnostic performance in differentiating WT from BCA compared with the other four classifiers. The AUC and accuracy were 0.861 and 0.94, with sensitivity, specificity and F-1 scores of 0.83, 0.90 and 0.91, respectively. The *p* value of the RF model in the Hosmer–Lemeshow test was 0412 (>0.05), so the calibration of the RF model was reliable. Analysis by DeLong’s test showed that the RF model had the highest AUC but that this value was not significantly different from those of the other models (*p>*0.05). The waterfall plot of the RF model in differentiating WT from BCA in the validation cohort is presented in **Figure 3E**. The ROC curve is shown in **Figure 4E**.

### PA *vs.* BCA

In the differentiation between PA and BCA, a total of 93 radiomics features were selected after being screened by the ICC and *t test*. Then, 10 features were finally selected by LASSO for building the radiomics models, and the best tuned regularization parameter lambda was 0.018. There were 2 first-order statistics features, 1 GLDM feature, 1 GLSZM feature and 6 wavelet features among the final selected features.

The radiomics model of the LR classifier obtained the best diagnostic performance between differentiating PA and BCA compared with the other four classifiers. However, the AUC and accuracy were only 0.602 and 0.68, yielding sensitivity, specificity and F-1 values of 0.66, 0.68 and 0.59, respectively. The *p* value of the LR model in the Hosmer–Lemeshow test was 0.357 (>0.05), so the calibration of the LR model was reliable. Analysis by DeLong’s test showed that the AUC of the LR model was not significantly different from those of the other four models (*p>*0.05). The waterfall plot of the LR model in differentiating PA from BCA in the validation cohort is presented in **Figure 3F**. The ROC curve is shown in **Figure 4F**.

The detailed selected features and coefficients of different radiomics models are shown in the **Supplementary Table**. The detailed diagnostic performance of all models is displayed in **Table 1**. The detailed results of DeLong’s test of the AUCs among the different models are shown in **Table 2**.

**Table 1:** Predictive performance of different models.

End-point	Models	AUC	95%CI	Accuracy	Sensitivity	Specificity	F1-score	H-L test (p-value)
MPGTs versus PA	RF	0.834	0.687-0.931	0.71	0.87	0.62	0.82	0.139
	SVM	0.816	0.666-0.918	0.67	0.88	0.54	0.80	0.051
	KNN	0.788	0.634-0.898	0.62	0.60	0.91	0.71	0.136
	LR	0.823	0.674-0.923	0.62	0.88	0.55	0.80	**0.003**
	Gnb	0.707	0.546-0.837	0.69	0.75	0.64	0.65	0.073
MPGTs versus WT	RF	0.887	0.751-0.963	0.79	0.75	0.83	0.88	0.433
	SVM	0.893	0.733-0.955	0.79	0.79	0.78	0.84	0.911
	KNN	0.872	0.732- 0.955	0.81	0.83	0.78	0.85	0.296
	LR	0.864	0.708-0.955	0.75	0.68	0.82	0.89	0.387
	Gnb	0.748	0.590-0.869	0.74	0.70	0.77	0.79	0.595
MPGTs versus BCA	RF	0.835	0.665-0.941	0.88	0.95	0.71	0.88	0.120
	SVM	0.838	0.669-0.943	0.82	0.84	0.85	0.86	0.052
	KNN	0.735	0.553-0.873	0.88	0.89	0.57	0.81	0.296
	LR	0.797	0.621-0.916	0.85	0.84	0.71	0.84	0.310
	Gnb	0.844	0.676-0.946	0.79	0.84	0.79	0.84	0.908
PA versus WT	RF	0.871	0.755-0.946	0.83	0.93	0.62	0.85	0.493
	SVM	0.878	0.762-0.950	0.83	0.87	0.79	0.86	0.299
	KNN	0.866	0.748-0.942	0.81	0.87	0.67	0.84	0.623
	LR	0.902	0.793-0.965	0.88	0.84	0.83	0.86	0.243
	Gnb	0.775	0.643-0.876	0.79	0.75	0.88	0.81	0.183
PA versus BCA	RF	0.570	0.368-0.772	0.68	0.75	0.50	0.57	**0.031**
	SVM	0.542	0.363-0.713	0.59	0.67	0.51	0.55	0.820
	KNN	0.591	0.410-0.756	0.65	0.66	0.50	0.54	0.869
	LR	0.602	0.421-0.765	0.68	0.66	0.68	0.59	0.357
	Gnb	0.587	0.406-0.753	0.65	0.63	0.59	0.56	0.378
WT versus BCA	RF	0.861	0.682- 0.960	0.94	0.83	0.90	0.91	0.412
	SVM	0.822	0.636-0.938	0.88	0.91	0.76	0.79	0.240
	KNN	0.786	0.595- 0.915	0.76	0.75	0.81	0.72	0.752
	LR	0.784	0.592-0.914	0.79	0.84	0.71	0.78	0.446
	Gnb	0.726	0.530-0.874	0.79	0.84	0.76	0.80	0.223

MPGTs, malignant parotid gland tumors; PA, pleomorphic adenoma; WT, warthin tumor; BCA, basal cell adenoma; SVM, support vector machine; RF, random forest; KNN, k-Nearest Neighbor; LR, logistic regression; Gnb, GaussianNB; AUC, area under the curve; CI, confidence interval; H-L, Hosmer–Lemeshow. Significant P values (<0.05) are in bold.

**Table 2:** Comparison of the performance of the different models with DeLong’s test.

Comparison	p-value	Comparison	p-value	Comparison	p-value
MPGTs *vs* PA		MPGTs *vs.* WT		MPGTs *vs.* BCA	
RF *vs*. SVM	0.609	RF *vs*. SVM	0.621	RF *vs*. SVM	0.961
RF *vs*. KNN	0.368	RF *vs*. KNN	0.601	RF *vs*. KNN	0.171
RF *vs*. LR	0.735	RF *vs*. LR	0.098	RF *vs*. LR	0.375
RF *vs*. Gnb	**0.021**	RF *vs*. Gnb	**0.015**	RF *vs*. Gnb	0.875
SVM *vs*. KNN	0.511	SVM *vs*. KNN	0.950	SVM *vs*. KNN	0.081
SVM *vs*. LR	0.748	SVM *vs*. LR	**0.022**	SVM *vs*. LR	0.603
SVM *vs*. Gnb	**0.005**	SVM *vs*. Gnb	**0.010**	SVM *vs*. Gnb	0.942
KNN *vs*. LR	0.452	KNN *vs*. LR	**0.015**	KNN *vs*. LR	0.305
KNN *vs*. Gnb	0.094	KNN *vs*. Gnb	**0.003**	KNN *vs*. Gnb	0.127
LR *vs*. Gnb	**0.008**	LR *vs*. Gnb	**< 0.001**	LR *vs*. Gnb	0.458
PA *vs* WT		PA *vs.* BCA		WT *vs.* BCA	
RF *vs*. SVM	0.807	RF *vs*. SVM	0.749	RF *vs*. SVM	0.197
RF *vs*. KNN	0.867	RF *vs*. KNN	0.756	RF *vs*. KNN	0.061
RF *vs*. LR	0.352	RF *vs*. LR	0.613	RF *vs*. LR	0.118
RF *vs*. Gnb	0.083	RF *vs*. Gnb	0.791	RF *vs*. Gnb	0.129
SVM *vs*. KNN	0.728	SVM *vs*. KNN	0.646	SVM *vs*. KNN	0.129
SVM *vs*. LR	0.353	SVM *vs*. LR	0.584	SVM *vs*. LR	0.529
SVM *vs*. Gnb	0.076	SVM *vs*. Gnb	0.651	SVM *vs*. Gnb	0.561
KNN *vs*. LR	0.339	KNN *vs*. LR	0.822	KNN *vs*. LR	0.599
KNN *vs*. Gnb	0.060	KNN *vs*. Gnb	0.961	KNN *vs*. Gnb	0.514
LR *vs*. Gnb	**0.019**	LR *vs*. Gnb	0.795	LR *vs*. Gnb	0.995

MPGTs, malignant parotid gland tumors; PA, pleomorphic adenoma; WT, warthin tumor; BCA, basal cell adenoma; SVM, support vector machine; RF, random forest; KNN, k-Nearest Neighbor; LR, logistic regression; Gnb, GaussianNB. Significant P values (<0.05) are in bold.

## Discussion

In this study, we provided a detailed analysis of the radiomics model based on noncontrast CT scans and advantageous machine learning classifiers that differentiate MPGTs, PA, WT and BCA. Our results revealed that noncontrast CT-based radiomics might help distinguish all parotid tumours with promising diagnostic results, except for the differentiation between PA and BCA. The classifier with the best diagnostic performance for each parotid tumour was different.

Radiomics uses mathematical calculations to identify invisible imaging features and then quantifies the different characteristics that parotid tumour tissues exhibit in radiological data to distinguish different parotid gland tumours (21). In our study, the highest AUCs in the comparisons of PA and MPGTs, WT and MPGTs, BCA and MPGTs, PA and WT, and BCA and WT were 0.834, 0.893, 0.844, 0.902 and 0.861, respectively. The diagnostic efficiency was promising and similar to that in previous studies. Zheng et al. extracted radiomics features from nonenhanced, arterial, and venous phase CT images and constructed LR-, SVM-, and RF-based radiomics models to differentiate between benign and malignant parotid tumours (22). They demonstrated that the model using SVM exhibited the best predictive accuracy, with an AUC of 0.844. Xu et al. extracted imaging features from noncontrast and contrast-enhanced CT images for differentiating between benign and malignant parotid gland tumours *via* multicentre cohorts (23). In their report, the accuracy of the SVM-based radiomics model reached 0.854. Xu et al. established a machine learning predictive model based on CT radiomics to improve the accuracy of differentiation among PA, WT and parotid carcinoma, with a total accuracy of 80.5% (24). All these studies used CT-based radiomics models to differentiate various parotid tumour types with promising performance. Unlike the abovementioned literature, we not only performed differentiation between benign and malignant tumours but also classified parotid tumours according to differences in pathological results, and various classifiers were used. Our study demonstrated that in addition to benign and malignant tumours, refined pathological types of parotid tumours could be stratified well by CT radiomics.

However, not all radiomics results are ideal. In our study, the nonenhanced CT-based radiomics model did not achieve good diagnostic performance in differentiating PA from BCA, and the highest AUC was only 0.602. It seemed that PA and BCA may not be effectively differentiated based on the noncontrast CT-based radiomics model alone. This result is similar to that in previous studies. Zheng et al. constructed radiomics models based on noncontrast CT for differentiating PA from BCA, and the AUCs of the models in the testing cohort with classifiers based on SVM, KNN, and LR were only 0.691, 0.612 and 0.652, respectively (25). This may be due to the pathological components of PA and BCA. The pathological structure of PA is complex and contains mixed components, such as glandular cells, myoepithelial cells, the parotid duct, mucus and cartilage-like tissue (26). In CT images, the density of the tumour was heterogeneous and may present cystic and necrosis. For BCA, there are four histological subtypes, namely, solid, trabecular, tubular, and membranous (27). Pathological composition varies by BCA histological subtype, which makes the radiomics features of BCA more complex. For the limited cases of BCA, we did not divide the BCA patients into different histological subtype groups. The mixed subtypes of BCA and high pathological heterogeneity of PA make it more difficult to differentiate them on noncontrast CT. Future radiomics models may need to incorporate additional CT-enhanced phases to refine model performance.

In addition, it should also be noted that among the selected radiomics features for predicting different tumours, most were transform-filtered features. The higher-order statistics performed by transform-filtered features can extract areas with increasingly coarse texture patterns more flexibly and thus have the potential to highlight more details in the original images (28). Among the transform-filtered features, wavelets were more valuable in our data analysis. The frequencies of wavelet features in the final selected features in the comparison of PA and MPGTs, WT and MPGTs, BCA and MPGTs, PA and WT, PA and BCA, and WT and BCA were 13/16, 11/14, 11/16, 8/18 6/10 and 9/15, respectively. Wavelet transforms can decompose image signals by using low- and high-pass filters and may amplify the heterogeneity information of texture features in radiological imaging, which is similar to previous studies. Jiang et al. reported that wavelet transformation can enhance CT texture features and may be used to effectively assess the grade of pulmonary lesions caused by COVID-19 (29). Regarding the best performance in discriminating an expansive from an infiltrative front in tumour growth, Granata et al. reported that wavelet transformation had the best performance in identifying tumour recurrence (30). This study suggests that in distinguishing different parotid gland tumours, the transform-filtered features, especially the wavelet transform-filtered features, may be more indicative of parotid tumour heterogeneity than other features (31).

In radiomics analysis, it is crucial to develop robust predictive models to select valid and appropriate modelling classifiers. Different classifiers mean different model algorithms and may lead to different diagnostic performances. Therefore, five frequently utilized machine learning classifiers were investigated in this study, namely, LR, KNN, RF, Gnb and SVM. LR is one of the most commonly used binary classification algorithms. The principle of KNN is that if most of the k-nearest samples near a sample belong to a certain category, the sample also belongs to this category. The advantages are that it is insensitive to outliers. RF is an ensemble algorithm with multiple decision trees. Its advantages include its high accuracy and that it does not easily result in overfitting, and its disadvantage is the large calculation. The mechanism of SVM is to build a decision boundary between two classes to predict labels from one or more feature vectors. SVM was powerful in analysing complex datasets but is also too complex to prevent overfitting. Finally, Gnb is a relatively simple algorithm but performs well on small-scale data. In our results, the classifier with the best diagnostic performance for each group was different. The classifiers with the highest AUCs in the comparisons of PA and MPGTs, WT and MPGTs, BCA and MPGTs, PA and WT, PA and BCA, and BCA and WT were RF, SVM, Gnb, LR, LR and RF, respectively. In addition, after analysis by DeLong’s test, in the comparisons of BCA and MPGTs, PA and BCA, and BCA and WT, there were no significant differences in AUC between the different classifiers. In the comparisons of MPGTs and PAs, MPGTs and WTs, and PAs and WTs, the AUCs of the best classifier were observed only to have significant differences with some of the classifiers. This was different from previous studies, which suggested that the performance of SVM was superior to that of other machine learning classifiers for total diagnostic accuracy (23, 25). We suggest that different classification models have their own advantages for different tasks. The performance of the radiomics model may depend more on the characteristics of the classifier algorithm and how well the classifiers match the model target tumour. Moreover, the different methods in radiomics feature extraction and selection would influence the final selected features and affect the diagnostic efficiency of models constructed with different classifiers. In our study, the results indicated that the key radiomics features among the different parotid tumours varied, so the selected classifier in the model with the best diagnostic efficacy was different. However, for the prediction efficiency of some parotid tumours, there seems to be no significant difference in the selection of classifiers. The results of our study could be a good reference in guiding the selection of the most appropriate classifiers for constructing different parotid gland tumour radiomics models.

There were several limitations in this study. First, potential selection bias may have occurred due to the retrospective nature of our study design. Second, the patients were enrolled from a single centre; thus, multicentre studies with much larger patient cohorts are necessary. Third, although our study included a large number of patients, the more detailed patient classification resulted in small numbers of cases in each group, especially the BCA group, so our study is still limited by the small number of samples in our dataset. Follow-up studies with larger sample sizes are needed. Fourth, to ensure that our results encompass different CT manufacturers, the CT-based radiomics features were from four different CT scanners. However, different scanning protocols, especially the fixed mA protocol, might affect the diagnostic performances of the radiomics features. Finally, we used the PyRadiomics package for feature extraction and image preprocessing in this study. Therefore, our results apply only to this package. Since other radiomics software packages may use different preprocessing filters, it is unclear whether our conclusions could apply to these radiomics packages. Regarding future research prospects, many machine learning radiomics studies have tried to predict early recurrence in different carcinomas after resection (32, 33), offering the possibility that radiomics models may also be used to predict recurrence in malignant parotid tumours after resection. Moreover, whether radiomics models could differentiate the inflammatory pathology of the parotid gland from neoplasms has rarely been discussed, and further studies are needed to research these topics.

## Conclusion

Based on this study, we propose using noncontrast CT-based radiomics features for the differential diagnosis of PA, WT, BCA and MPGT, as they show good predictive performance for all comparisons except for that of PA and BCA. Our findings suggest that noncontrast CT radiomics analysis can be used as an additional tool to support radiologists in their decision-making in distinguishing different parotid gland tumours.

## Data availability statement

The raw data supporting the conclusions of this article will be made available by the authors, without undue reservation.

## Ethics statement

Written informed consent was obtained from the individual(s) for the publication of any potentially identifiable images or data included in this article.

## Author contributions

All authors contributed to the study conception and design. Study concepts and design were performed by YL, WX. Material preparation, data collection and analysis were performed by YL, QL, ZZ, SW, and HL, JQ made substantial contributions to data acquisition and interpretation. The first draft of the manuscript was written by YL and all authors commented on previous versions of the manuscript. All authors contributed to the article and approved the submitted version.
