# Supplementary material for: CT-based radiomics with various classifiers for histological differentiation of parotid gland tumors
Source: Front Oncol. 2023 Mar 10;13:1118351. doi: 10.3389/fonc.2023.1118351 (PMC10036756; doi:10.3389/fonc.2023.1118351)
Supplement: Supplementary file 1 [file Table_1.docx]

**Supplementary Table.** The coefficients of features in the model.

| Model | Features | Coef in Model |
| --- | --- | --- |
| MPGTs versus PA | original_firstorder_InterquartileRange | -4.745490e-02 |
|  | original_glcm_Imc2 | -7.835839e-02 |
|  | gradient_glrlm_RunLengthNonUniformityNormalized | -3.339322e-02 |
|  | wavelet-LLH_firstorder_Kurtosis | -5.245987e-02 |
|  | wavelet-LHL_glcm_Autocorrelation | -8.152828e-03 |
|  | wavelet-LHH_glcm_InverseVariance | 1.209389e-02 |
|  | wavelet-HLL_glszm_GrayLevelNonUniformity | -1.800011e-02 |
|  | wavelet-HLH_glcm_InverseVariance | 3.258772e-03 |
|  | wavelet-HLH_glszm_SmallAreaEmphasis | -1.737908e-02 |
|  | wavelet-HHL_glcm_Correlation | -2.300917e-02 |
|  | wavelet-HHL_glszm_GrayLevelNonUniformity | -1.636463e-03 |
|  | wavelet-HHH_firstorder_Kurtosis | -4.607604e-02 |
|  | wavelet-HHH_glcm_InverseVariance | 9.487977e-03 |
|  | wavelet-HHH_glcm_MCC | -1.726725e-03 |
|  | wavelet-HHH_glszm_SmallAreaLowGrayLevelEmphasis | -2.012206e-02 |
|  | wavelet-LLL_glcm_JointAverage | -2.041646e-03 |

*PA, pleomorphic adenoma; MPGTs, malignant parotid gland tumors*

| Model | Features | Coef in Model |
| --- | --- | --- |
| MPGTs versus WT | original_shape_Flatness | -0.093554 |
|  | exponential_glrlm_ShortRunLowGrayLevelEmphasis | -0.129407 |
|  | logarithm_firstorder_Kurtosis | 0.050610 |
|  | wavelet-LLH_glcm_Correlation | -0.024645 |
|  | wavelet-LLH_glszm_SmallAreaLowGrayLevelEmphasis | 0.048252 |
|  | wavelet-LHL_glszm_GrayLevelNonUniformityNormalized | 0.043633 |
|  | wavelet-HLL_firstorder_Kurtosis | -0.025445 |
|  | wavelet-HLH_glcm_Correlation | -0.007951 |
|  | wavelet-HLH_gldm_DependenceVariance | -0.060299 |
|  | wavelet-HLH_glszm_GrayLevelNonUniformity | -0.025163 |
|  | wavelet-HLH_glszm_LowGrayLevelZoneEmphasis | 0.029177 |
|  | wavelet-HHL_firstorder_Kurtosis | -0.002125 |
|  | wavelet-HHL_glcm_InverseVariance | 0.035548 |
|  | wavelet-LLL_firstorder_Median | 0.018380 |

*WT, warthin tumor; MPGTs, malignant parotid gland tumors*

| Model | Features | Coef in Model |
| --- | --- | --- |
| MPGTs versus BCA | original_shape_Sphericity | 0.055241 |
|  | original_glcm_MCC | -0.004910 |
|  | original_glrlm_GrayLevelNonUniformityNormalized | 0.024120 |
|  | exponential_firstorder_InterquartileRange | -0.026885 |
|  | exponential_glrlm_RunLengthNonUniformity | -0.003769 |
|  | wavelet-LLH_gldm_DependenceNonUniformityNormalized | 0.021330 |
|  | wavelet-LLH_glszm_SmallAreaLowGrayLevelEmphasis | 0.027197 |
|  | wavelet-LHL_glrlm_LongRunHighGrayLevelEmphasis | -0.008646 |
|  | wavelet-LHL_ngtdm_Busyness | 0.069454 |
|  | wavelet-HLL_firstorder_Kurtosis | -0.002762 |
|  | wavelet-HLH_glcm_InverseVariance | 0.059459 |
|  | wavelet-HLH_glszm_SmallAreaEmphasis | -0.019161 |
|  | wavelet-HHL_glszm_GrayLevelNonUniformityNormalized | 0.015261 |
|  | wavelet-HHL_glszm_LowGrayLevelZoneEmphasis | 0.029425 |
|  | wavelet-LLL_firstorder_Minimum | 0.027829 |
|  | wavelet-LLL_gldm_LargeDependenceLowGrayLevelEmphasis | 0.026355 |

*BCA, basal cell adenoma; MPGTs, malignant parotid gland tumors*

| Model | Features | Coef in Model |
| --- | --- | --- |
| PA versus WT | original_shape_Flatness | -0.134024 |
|  | original_shape_SurfaceArea | -0.082960 |
|  | original_firstorder_InterquartileRange | 0.036975 |
|  | original_glcm_MaximumProbability | -0.084291 |
|  | gradient_glszm_ZonePercentage | -0.023137 |
|  | logarithm_firstorder_Skewness | -0.034360 |
|  | logarithm_firstorder_TotalEnergy | 0.044789 |
|  | logarithm_gldm_DependenceVariance | 0.027711 |
|  | squareroot_gldm_DependenceVariance | 0.047318 |
|  | squareroot_gldm_LargeDependenceHighGrayLevelEmphasis | 0.011053 |
|  | wavelet-LLH_firstorder_TotalEnergy | -0.035536 |
|  | wavelet-LLH_glcm_Imc2 | -0.039357 |
|  | wavelet-LHH_glszm_SmallAreaLowGrayLevelEmphasis | 0.016066 |
|  | wavelet-HLL_glcm_DifferenceEntropy | -0.020359 |
|  | wavelet-HLL_glszm_ZonePercentage | -0.046258 |
|  | wavelet-HHL_gldm_DependenceVariance | -0.109675 |
|  | wavelet-LLL_firstorder_RootMeanSquared | 0.104697 |
|  | wavelet-LLL_glcm_ClusterShade | -0.037637 |

*PA, pleomorphic adenoma; WT, warthin tumor*

| Model | Features | Coef in Model |
| --- | --- | --- |
| PA versus BCA | diagnostics_Image-original_Mean | 0.005722 |
|  | diagnostics_Image-original_Minimum | 0.074034 |
|  | original_gldm_DependenceNonUniformityNormalized | 0.001419 |
|  | original_glszm_GrayLevelNonUniformityNormalized | 0.020698 |
|  | wavelet-LHL_firstorder_90Percentile | -0.100892 |
|  | wavelet-HLL_firstorder_Maximum | -0.006810 |
|  | wavelet-HLL_glcm_Id | 0.056165 |
|  | wavelet-HLL_glcm_InverseVariance | -0.018324 |
|  | wavelet-HLH_glcm_InverseVariance | 0.045238 |
|  | wavelet-LLL_firstorder_Minimum | 0.028447 |

*PA, pleomorphic adenoma; BCA, basal cell adenoma*

| Model | Features | Coef in Model |
| --- | --- | --- |
| WT versus BCA | original_shape_Flatness | 0.146327 |
|  | original_firstorder_Skewness | 0.035254 |
|  | original_firstorder_Uniformity | 0.018927 |
|  | gradient_glszm_ZonePercentage | 0.073572 |
|  | logarithm_glcm_Imc2 | 0.006287 |
|  | square_firstorder_Median | -0.041404 |
|  | wavelet-LLH_glcm_MCC | 0.087259 |
|  | wavelet-LLH_gldm_DependenceVariance | -0.032916 |
|  | wavelet-LHL_firstorder_90Percentile | -0.029381 |
|  | wavelet-LHL_glcm_Correlation | 0.021740 |
|  | wavelet-LHL_ngtdm_Busyness | 0.072592 |
|  | wavelet-HLL_glszm_SizeZoneNonUniformityNormalized | -0.033317 |
|  | wavelet-HHH_glcm_ClusterShade | -0.028323 |
|  | wavelet-LLL_firstorder_TotalEnergy | -0.026200 |
|  | wavelet-LLL_glszm_SmallAreaEmphasis | -0.087331 |

*WT, warthin tumor; BCA, basal cell adenoma*
